# Puberty timing associated with obesity and central obesity in Chinese Han girls

**DOI:** 10.1186/s12887-018-1376-4

**Published:** 2019-01-03

**Authors:** Qiguo Lian, Yanyan Mao, Shan Luo, Shucheng Zhang, Xiaowen Tu, Xiayun Zuo, Chaohua Lou, Weijin Zhou

**Affiliations:** 1 NHC Key Lab. of Reproduction Regulation (Shanghai Institute of Planned Parenthood Research), School of Public Health, Fudan University, 779 Laohumin Road, Shanghai, 200237 China; 2 West China Second University Hospital, Sichuan University, Chengdu, Sichuan China; 3 National Research Institute for Family Planning, Beijing, China; 4 Key Laboratory of Birth Defects and Reproductive Health of National Health and Family Planning Commission (Chongqing Population and Family Planning Science and Technology Research Institute), Chongqing, China

**Keywords:** Obesity, Central obesity, Puberty timing, Tanner stage

## Abstract

**Background:**

There is growing scientific evidence supports a link between increased childhood adiposity and early onset of puberty in girls worldwide in recent decades. However, the data from Chinese girls remain ambiguous. The aims of this study were to estimate the puberty milestones and examine attainment of puberty associated with obesity and central obesity in Chinese Han schoolgirls.

**Methods:**

The cross-sectional school-based study examined 2996 Han schoolgirls aged 9 to 19 years from 6 provinces in China. Trained clinicians assessed  the girls for height, weight, waist circumference, Tanner stages of breast and pubic hair development, and menarcheal status. We classified girls as normal weight, overweight, or obese based on BMI, and as normal weight or central obese based on the waist-height ratio, then estimated and compared median age at a given Tanner stage or greater by weight class using Probit models.

**Results:**

The median age at menarche was 12.36 years. The median ages at breast stages(B) 2 through 5 were 10.03, 11.38, 13.39, and 15.79 years, respectively, and at pubic hair stages(PH) 2 through 5 were 11.62, 12.70, 14.38, and 16.92 years, respectively. Girls from urban areas experienced menarche, B3 and B4 stages, and PH3 through PH5 stages earlier. Girls with central obesity and overweight/obesity reached puberty earlier at almost every Tanner stage of breast and pubic hair than normal girls. Girls with obesity developed PH2 and PH3 earlier than their overweight peers. However, we did not find any significant differences between girls with overweight and obesity at all stages of breast development.

**Conclusions:**

Childhood obesity, including both overweight/obesity and central obesity, is associated with earlier attainment of puberty in Chinese Han schoolgirls.

## Background

Puberty is a period characterized by a growth spurt and rapid development of secondary sexual characteristics (breast budding, pubic hair growth and menarche in girls). The first visible evidence of puberty in girls is marked by thelarche, as developed by Tanner stages [1] . Altered timing of puberty has significant clinical implications in pediatrics for the treatment of individual children. However, much of data on timing of puberty in girls focused on the age of menarche, which is easier to measure and least affected by observation errors [2] .

Globally, most of the studies on timing of puberty were conducted using clinical samples [2–5]. However, large scale, population-based epidemiologic data on puberty development is very important, for both pediatricians in precocious puberty and researchers focusing on the timing of puberty [6] . The secular trend to earlier pubertal development in children and adolescents has been reported by many researchers, which was commonly ascribed by the improvement of nutritional and general health conditions [2, 6, 7] . Yet it is worth noting that, during recent decades, several investigators noted a halt or even reversal in this trend in different countries, including China [6, 8–10] .

Age of pubertal development has a great impact on health conditions, and early puberty might translate into increased risk for adult-onset diabetes, breast cancer, and all-cause mortality [7, 11, 12] . The relationship between childhood adiposity, as assessed by body mass index (BMI), and earlier onset of puberty in girls has been reported in multiple studies [13–15] . Existing Chinese studies on the relationship between obesity and pubertal attainment in girls were limited by lack of multiple measures of puberty development and different criteria  of grouping childhood adiposity [6] . Many epidemiologic studies used self-assessment of pubertal maturation, given that individual assessments by clinicians are time-consuming and expensive. Although central fat, measured by waist circumference (WC) and the waist-height ratio (WHtR), raises the risk for metabolic and cardiovascular complications in adolescents, it has been rarely used as the indicator of childhood adiposity in most findings [16] .

To bridge the gap, we conducted a national cross-sectional study in China, to explore the association between body weight (overweight/obesity and central obesity) and attainment of puberty (breast development, pubic hair growth, and menarche) in Chinese Han schoolgirls. And we also wanted to estimate the ages of pubertal milestones for girls with and without obesity.

## Methods

### Study sites and participants

A school-based cross-sectional study was conducted among 26 sampled schools from 6 provinces in China during November 2012 to April 2013. Multistage cluster sampling strategy was introduced to ensure a representative sample. First, given the heavily unbalanced development of China, six provinces were selected to represent the six geographical regions in China Mainland: Hebei (North China), Shandong (East China), Heilongjiang(Northeast China), Guangxi(South Central China), Sichuan(Southwest China) and Shaanxi(Northwest China). Second, three public schools (one primary school, one senior high school, and one junior high school) were sampled respectively from urban and rural areas of one moderately developed city in each selected province. Finally, two classes were randomly chosen from grade 5 to grade 12 respectively. All female students from those classes were recruited to the examination if they had no major-medical diseases recorded in the school clinic medical cards, including developmental conditions or chronic diseases. Totally 3463 healthy schoolgirls (Han girls 2996, minority girls 467) were examined voluntarily in school clinics by a team of well-trained female clinicians. In the present study, only 2996 Han schoolgirls were included in the final analysis, considering that the sample size of minority girls was not large enough and the onset of puberty may be related to ethnicity [17].

### Ethical issues

We provided information sheets about the study to all the students and their teachers and guardians 1 week before the clinical examination, the sheets highlighted that the examination was anonymous and non-invasive, the students were free to quit the examination if they felt uncomfortable. Written informed consent was obtained from the guardians of all students. Verbal consent was obtained from students before the examination. This study was reviewed and approved by the institutional review board of Shanghai Institute of Planned Parenthood Research (2012–01).

### Measures

Anthropometric measures were taken on standing participants wearing light clothing and without shoes using standard techniques. Height was measured by portable calibrated stadiometers. WC was measured midway between the lateral lower rib margin and the iliac crest using a non-stretchable measurement tape. The measurements of the height and WC were in centimeters (cm) to the nearest 0.1 cm. Weight was measured to the nearest 0.5 kg.

Sexual maturity was evaluated with Tanner’s five stages (stage 1 represents immaturity, and stage 5 indicates full maturity). Breast development (B) was evaluated by both inspection and palpation, to avoid the misclassification and overestimation in girls with obesity by routine visual assessment only [18], Tanner stage 2 for breast development is marked as B2. Pubic hair growth (PH) was rated by direct observation at clinical examination, Tanner stage 2 for pubic hair growth is marked as PH2. If Tanner stage fell between two stages, the clinicians rated the girl as the lower stage. Our estimates of puberty timing, hence, would be conservative. Besides, the status quo method was used to evaluate the median age at menarche. Before the clinical examination, clinical assistants asked every girl’s birth date and menarcheal status (yes/no), given that such information produces more precise estimates than complex recall methods achieve [19] .

### Study definitions

#### Age

Age is a continuous variable. In this study, we defined age (years) as the number of days between birth date and examination date divided by 365.25. The age was categorized by function int(age) when necessary. For example, the 12-year-old group contained girls aged 12.00 to 12.99.

#### Weight status

BMI was computed as weight (kg) divided by height squared (m^2^). Given that Chinese sex-age-specific BMI references recommended by Working Group on Obesity in China(WGOC) is similar with World Health Organization BMI references, and can reflect better the body composition of Chinese and other Asian populations [20],we classified the female students into 3 categories: normal weight, overweight and obese using the WGOC BMI cutoffs [20]. WHtR was computed as the WC in meters divided by the height in meters. We marked the female students as central obesity by 2 references: WHtR≥0.5 [21] and WC ≥ 90th percentile [22].

#### Main outcome measures

We used breast stages, pubic hair stages, and menarche as the main outcome measures. According to the breast development, we classified the female students as 1) B2 and greater versus B1; 2) B3 and greater versus B1 or B2; 3) B4 and greater versus B1, B2 or B3; 4) B5 and greater versus B1,B2, B3 or B4. We took the same approach to the categorization of the pubic hair growth. We listed the median age at menarche by WHtR, WC, weight status, and rural/urban residence.

#### Statistical analysis

We calculated the percentage of girls by age group, place, weight status, and pubertal stages. We conducted probit regression models for menarche, breast and pubic hair stages to predict the probability of reaching each landmark by age [23] . We estimated the median age and its 95% confidence intervals (CIs), and compared median age at menarche and pubertal stages for female students of different groups, i.e. 1) urban versus rural, 2) central obese versus normal, 3) normal weight versus overweight, normal weight versus obese, and overweight versus obese. We considered statistical significance at *p* < 0.05 and did not perform statistical adjustment for multiple comparisons, as has been recommended in the statistical literature [24]. All the analyses were conducted with Stata/SE 14.2 (StataCorp, College Station, TX, USA) [25] .

## Results

Table 1 shows the descriptive characteristics of the girls in our study. A total of 2996 female Han Chinese girls aged 8.00–19.99 years participated in this study. Among them, 47.36% (*n* = 1419) of the girls came from urban, and 52.64% (*n* = 1577) lived in rural. According to the cutoffs of Chinese sex-age-specific BMI reference recommended by WGOC, more than 85% of the female students (85.88%, *n* = 2573) were normal weight, 8.68% (*n* = 260) were overweight, and 5.44% (*n* = 163) were obese. The proportion of central obese was 14.69%  using 0.5 cutoffs of WHtR, and the estimate was similar to the reference of 90th WC percentiles. Table 1 also displays the distribution of Tanner Stage of breast development and pubic hair development. 2169 girls (72.40%) experienced menarche.

**Table 1:** Descriptive Characteristics of the Population by Demographics and Development

Characteristic	n	%
Place		
Urban	1419	47.36
Rural	1577	52.64
Weight Status		
Normal weight	2573	85.88
Overweight	260	8.68
Obese	163	5.44
WHtR		
< 0.5	2556	85.31
≥ 0.5	440	14.69
WC		
< 90th	2670	89.12
≥ 90th	326	12.88
Breast Development		
B1	201	6.71
B2	311	10.38
B3	705	23.53
B4	829	27.67
B5	950	31.71
Pubic Hair Growth		
PH1	578	19.29
PH2	376	12.55
PH3	603	20.13
PH4	791	26.40
PH5	648	21.63
Menarcheal status		
Yes	2169	72.40
No	827	27.60

Abbreviations: B, Breast stage; PH, Pubic hair stage; WHtR, Waist to height ratio; WC, Waist circumference

Table 2 displays the median age at Tanner B2 or greater through B5 or greater according to obese status and demographics. The median age was 10.03 years at B2, 11.38 years at B3, 13.39 years at B4 and 15.79 years at B5. We found that the median age of girls marked as central obesity by either WHtR or WC were earlier than of normal girls across all stages of breast development, and all the differences were statistically significant. Similarly, compared with overweight or obesity group, there was evidence of a trend of later median age in normal weight group across all stages. However, we did not find any significant difference in timing of puberty between overweight and obesity. Besides, the median ages of urban girls were earlier than those of rural girls in all stages except in B5 or greater (15.70 years [urban] versus 15.88 years [rural]; *P* = 0.094).

**Table 2:** Median Age at Tanner Stages for Breast Development by Demographics

	≥B2 Median Age (95% CI)	*P* value	≥B3 Median Age (95% CI)	*P* value	≥B4 Median Age (95% CI)	*P* value	≥B5 Median Age (95% CI)	*P* value
Overall	10.03 (9.80, 10.27)	–	11.38(11.26,11.49)	–	13.39(13.29,13.49)	–	15.79(15.69,15.90)	–
Place								
Urban	10.19(9.91,10.46)		11.26(11.11,11.41)		13.25(13.11,13.39)		15.70(15.54,15.85)	
Rural	9.85(9.45,10.25)	0.187	11.50(11.33,11.66)	0.043	13.51(13.37,13.64)	0.010	15.88(15.73,16.03)	0.094
WHtR								
< 0.5	10.12(9.89,10.5)		11.43(11.31,11.55)		13.48(13.37,13.59)		15.90(15.79,16.02)	
≥ 0.5	9.00(7.60,10.41)	0.007	11.00(10.59,11.41)	0.013	12.92(12.70,13.14)	< 0.001	15.22(14.92,15.51)	< 0.001
WC								
< 90th	10.05(9.81,10.29)		11.41(11.29,11.53)		13.48(13.37,13.58)		15.92(15.80,16.03)	
≥ 90th	8.44(4.23,12.65)	0.052	10.65(9.79,11.52)	0.001	12.77(12.48,13.07)	< 0.001	15.00(14.68,15.31)	< 0.001
BMI								
Normal weight	10.25(10.03,10.47)		11.54(11.42,11.66)		13.48(13.38,13.59)		15.86(15.75,15.97)	
Overweight	8.64(6.85,10.43)	< 0.001^a^	10.37(9.71,11.03)	< 0.001^a^	12.83(12.54,13.13)	< 0.001^a^	15.40(14.96,15.83)	0.019^a^
Obese	8.60(6.54,10.65)	0.002^b^	10.66(10.00,11.31)	0.001^b^	12.93(12.51,13.36)	0.015^b^	15.16(14.67,15.66)	0.004^b^
		0.975^c^		0.570^c^		0.686^c^		0.495^c^

*Abbreviations*: *B* Breast stage, *BMI* Body mass index, *CI* Confidence interval, *WHtR* Waist to height ratio, *WC* Waist circumference

^a^*P* value of comparison between overweight and normal weight

^b^ P value of comparison between obesity and normal weight

^c^*P* value of comparison between overweight and obesity

Table 3 demonstrates differences in median age at Tanner PH2 or greater through PH5 or greater according to obese status and demographics. The median age was 11.62 years at PH2, 12.70 years at PH3, 14.38 years at PH4 and 16.92 years at PH5. These age differences for central obese/normal group, urban/rural area, overweight/normal weight and obese/normal weight group were consistent across all stages of pubic hair development. Compared with the obese group, there was evidence for later median age in the overweight group for PH2 or greater (11.31 years [overweight] versus 10.57 years [obese], *P* = 0.021) and PH3 or greater (12.49 years [overweight] versus 11.93 years [obese], *P* = 0.031).

**Table 3:** Median Age at Tanner Stages for Pubic Hair Development by Demographics

	≥PH2 Median Age (95% CI)	*P* value	≥PH3 Median Age (95% CI)	*P* value	≥PH4 Median Age (95% CI)	*P* value	≥PH5 Median Age (95% CI)	*P* value
Overall	11.62(11.51,11.73)	–	12.70(12.61,12.80)	–	14.38(14.28,14.48)	–	16.92(16.78,17.06)	–
Place								
Urban	11.53(11.38,11.68)		12.38(12.24,12.53)		13.98(13.84,14.12)		16.61(16.43,16.78)	
Rural	11.70(11.55,11.85)	0.132	12.97(12.85,13.10)	< 0.001	14.71(14.57,14.86)	< 0.001	17.25(17.02,17.48)	< 0.001
WHtR								
< 0.5	11.68(11.56,11.79)		12.76(12.66,12.86)		14.48(14.38,14.60)		16.98(16.84,17.13)	
≥ 0.5	11.25(10.91,11.60)	0.008	12.30(12.00,12.60)	0.001	13.82(13.57,14.08)	< 0.001	16.55(16.15,16.94)	0.029
WC								
< 90th	11.68(11.57,11.79)		12.77(12.67,12.87)		14.49(14.38,14.59)		16.98(16.84,17.13)	
≥ 90th	9.83(8.37,11.30)	< 0.001	11.52(10.84,12.20)	< 0.001	13.63(13.31,13.94)	< 0.001	16.49(16.05,16.92)	0.028
BMI								
Normal weight	11.76(11.64,11.87)		12.79(12.69,12.90)		14.48(14.37,14.59)		17.01(16.87,17.16)	
Overweight	11.31(10.96,11.66)	0.021^a^	12.49(12.16,12.82)	0.086^a^	13.71(13.37,14.04)	< 0.001^a^	16.03(15.59,16.47)	< 0.001^a^
Obese	10.57(10.00,11.14)	< 0.001^b^	11.93(11.54,12.31)	< 0.00^b^	13.81(13.45,14.18)	0.003^b^	16.29(15.52,17.06)	0.022^b^
		0.021^c^		0.031^c^		0.631^c^		0.535^c^

*Abbreviations*: *BMI* Body mass index, *CI* Confidence interval, *PH* Pubic hair stage, *WHtR* Waist to height ratio, *WC* Waist circumference

^a^*P* value of comparison between overweight and normal weight

^b^ P value of comparison between obesity and normal weight

^c^ P value of comparison between overweight and obesity

The median age at menarche was 12.36 years for Chinese Han girls. As illustrated in Table 4, there was evidence of a trend for earlier median age of onset in urban (vs. rural) area, central obese (vs. normal group) group, overweight (vs. normal weight) and obese (vs. normal weight) group. We did not observe any significant difference in median age between overweight and obese subgroups.

**Table 4:** Median Age at Menarche by Demographics

	Menarche	*P* value
	Median Age (95% CI)	
Overall	12.36(12.27,12.45)	–
Place		
Urban	12.17(12.04,12.30)	
Rural	12.52(12.40,12.65)	< 0.001
WHtR		
< 0.5	12.47(12.38,12.57)	
≥ 0.5	11.70(11.43,11.97)	< 0.001
WC		
< 90th	12.44(12.35,12.54)	
≥ 90th	11.48(11.04,11.93)	< 0.001
BMI		
Normal weight	12.51(12.42,12.61)	
Overweight	11.75(11.50,12.00)	< 0.001^a^
Obese	11.44(11.04,11.84)	< 0.001^b^
		0.175^c^

*Abbreviations*: *BMI* Body mass index, *CI* confidence interval, *WHtR* Waist to height ratio, *WC* Waist circumference

^a^ P value of comparison between overweight and normal weight

^b^ P value of comparison between obesity and normal weight

^c^ P value of comparison between overweight and obesity

## Discussion

This study examined the association between timing of puberty staging (breast development, pubic hair development, and menarche) and obesity (overweight/obesity and central obesity) with a national sample of Han schoolgirls using a cross-sectional design. The measurements on puberty staging and obesity from clinical examinations were detailed and objective. This study adds unique and valuable data in Asian girls because existing large studies have focused more on European, African American or Hispanic descent populations [5, 15, 18, 19].

Our findings add to evidence of earlier puberty (PH2 and PH3) for obese compared with normal or overweight girls. Also, there was suggestive evidence of later puberty (PH4 and PH5) for obese compared with overweight girls, although the differences in timing of PH4 and PH5 between the subgroups were non-significant. However, the differences of breast development and menarche were not significant between overweight and obese subgroups.

Considering that the effect of estrogen on the linear growth in humans is biphasic, low dose promotes, but high dose suppresses the linear growth [26], it was hypothesized that excessive aromatase activity in adipose tissue produces increased level of estrogen, eventually results in possible delayed puberty. We found that compared with overweight girls, obese girls started PH2 earlier and attained PH4 and PH5 later, which indicates that excessive estrogen production in obese girls may inhibit the puberty process (PH4 and PH5) for obese, but not overweight, girls. Consistent with previous study conducted among boys [27], our findings suggest that there is also a nonlinear relationship between pubic hair development and body fat for girls, which provides valuable insight into the complex relationship between puberty timing, overweight and obesity.

Note that it’s difficult to compare the results directly with previous studies, because the weight status data based on BMI, in most of the studies, were not divided into normal weight, overweight and obese sub groups [28, 29]. In a national survey, Ying et al. found that an elevated BMI was associated with significantly earlier attainment of breast and menarche [6] .

In our study, girls with central obesity, measured by either WC or WHtR, achieved all stages of breast and pubic hair development earlier than girls without central obesity. In adolescents, as in adults, central fat increases the risk for metabolic syndrome (dyslipidemia and insulin resistance) [30, 31]. WC and WHtR in children are better indicators of distribution of body fat than BMI [32, 33], easily measurable WC may be useful to help to identify vulnerable children [34] . Our findings suggest that WC and WHtR can also server as obesity indicators to identify girls at risk of early pubertal onset.

In the present study, urban girls reached menarche earlier than girls living in rural places (12.17 years vs. 12.52 years, *p* < 0.001) possibly because urban residents were more likely than rural residents to have access to high-quality food [6] . However, we did not find the age differences of B2 and PH2 between urban and rural girls, which implies that breast and pubic hair development may be less sensitive than menarche to nutrition status [6] . Body size parameters, such as BMI and body fat distribution are strongly correlated with the age at menarche. In our study, the median age of menarche of central obese and overweight/obese girls was earlier than that of normal girls. Frisch et al. proposed a critical body weight and weight gain to trigger the onset of puberty [35] . Childhood adiposity may be considered as one of the predictors for the early occurrence of menarche [36] .

### Strengths and limitations

This study has several strengths. First, the present study included the subjects from a multi-provincial school-based population, which allows us to investigate the associations at the national level. Second, we rated puberty by triple objective indicators, assessed by clinicians, which is rare in large scale epidemiological studies. In addition, all the examinations were performed by the same group of reproductive endocrinologists, which guaranteed the comparability of results from diverse sites. The main limitation is that the nature of cross-sectional study cannot determine the direction of causality between pubertal development and obesity, obesity can both cause and result from pubertal development. Despite that, our study can help generate hypotheses to be explored using longitudinal studies. Second, we did not measure hormonal indictors, including estrogen, sex hormone-binding globulin, luteinizing hormone and follicle-stimulating hormone, which may confound our results because hormone could influence both puberty timing and weight status [37]. Third, we failed to collect the information of the parents’ pubertal development, especially maternal history of precocious puberty, which may influence the pubertal development of their offspring. Fourth, participants were not sampled with multistage probability technique, which lowered its representativeness to a certain extent. Last, we may overestimate median age at Tanner B2 or greater given than we did not included children aged 6–7 years.

## Conclusion

In conclusion, childhood obesity, including overweight/obesity and central obesity, is associated with earlier attainment of puberty at almost every pubertal stage of breast, pubic hair and menarche among Chinese Han schoolgirls. Our data support the potential nonlinear relationship between time of puberty and body fat in girls, which suggests that more studies are needed to explore the biological mechanisms behind.

## Abbreviations

B: Breast stage
BMI: Body mass index
CI: Confidence interval
PH: Pubic hair stage
WC: Waist circumference
WHtR: Waist to height ratio

## References

[1] Marshall WA, Tanner JM (1969). Variations in pattern of pubertal changes in girls. Arch Dis Child.

[2] Ong KK, Ahmed ML, Dunger DB (2006). Lessons from large population studies on timing and tempo of puberty (secular trends and relation to body size): the European trend. Mol Cell Endocrinol.

[3] Juul A, Teilmann G, Scheike T, Hertel NT, Holm K, Laursen EM, Main KM, Skakkebaek NE (2006). Pubertal development in Danish children: comparison of recent European and US data. Int J Androl.

[4] Aksglaede L, Sorensen K, Petersen JH, Skakkebaek NE, Juul A (2009). Recent decline in age at breast development: the Copenhagen puberty study. Pediatrics.

[5] Euling SY, Herman-Giddens ME, Lee PA, Selevan SG, Juul A, Sorensen TI, Dunkel L, Himes JH, Teilmann G, Swan SH (2008). Examination of US puberty-timing data from 1940 to 1994 for secular trends: panel findings. Pediatrics.

[6] Sun Y, Tao FB, Su PY, Mai JC, Shi HJ, Han YT, Wang H, Lou XM, Han J, Liu J (2012). National estimates of the pubertal milestones among urban and rural Chinese girls. J Adolesc Health.

[7] Parent AS, Rasier G, Gerard A, Heger S, Roth C, Mastronardi C, Jung H, Ojeda SR, Bourguignon JP (2005). Early onset of puberty: tracking genetic and environmental factors. Horm Res.

[8] Cole TJ (2000). Secular trends in growth. Proc Nutr Soc.

[9] Atay Z, Turan S, Guran T, Furman A, Bereket A (2011). Puberty and influencing factors in schoolgirls living in Istanbul: end of the secular trend?. Pediatrics.

[10] Papadimitriou A (2016). The evolution of the age at menarche from prehistorical to modern times. J Pediatr Adolesc Gynecol.

[11] Lakshman R, Forouhi N, Luben R, Bingham S, Khaw K, Wareham N, Ong KK (2008). Association between age at menarche and risk of diabetes in adults: results from the EPIC-Norfolk cohort study. Diabetologia.

[12] Jacobsen BK, Heuch I, Kvale G (2007). Association of low age at menarche with increased all-cause mortality: a 37-year follow-up of 61,319 Norwegian women. Am J Epidemiol.

[13] Biro FM, Greenspan LC, Galvez MP, Pinney SM, Teitelbaum S, Windham GC, Deardorff J, Herrick RL, Succop PA, Hiatt RA (2013). Onset of breast development in a longitudinal cohort. Pediatrics.

[14] Rosenfield RL, Lipton RB, Drum ML (2009). Thelarche, pubarche, and menarche attainment in children with normal and elevated body mass index. Pediatrics.

[15] Kaplowitz PB, Slora EJ, Wasserman RC, Pedlow SE, Herman-Giddens ME (2001). Earlier onset of puberty in girls: relation to increased body mass index and race. Pediatrics.

[16] Bacopoulou F, Efthymiou V, Landis G, Rentoumis A, Chrousos GP (2015). Waist circumference, waist-to-hip ratio and waist-to-height ratio reference percentiles for abdominal obesity among Greek adolescents. BMC Pediatr.

[17] Song Y, Ma J, Agardh A, Lau PW, Hu P, Zhang B (2015). Secular trends in age at menarche among Chinese girls from 24 ethnic minorities, 1985 to 2010. Glob Health Action.

[18] Kaplowitz PB, Oberfield SE (1999). Reexamination of the age limit for defining when puberty is precocious in girls in the United States: implications for evaluation and treatment. Drug and therapeutics and executive committees of the Lawson Wilkins pediatric Endocrine Society. Pediatrics.

[19] Chumlea WC, Schubert CM, Roche AF, Kulin HE, Lee PA, Himes JH, Sun SS (2003). Age at menarche and racial comparisons in US girls. Pediatrics.

[20] Ji CY, Working Group on Obesity in C (2005). Report on childhood obesity in China (1)--body mass index reference for screening overweight and obesity in Chinese school-age children. Biomed Environ Sci.

[21] Yan W, Bingxian H, Hua Y, Jianghong D, Jun C, Dongliang G, Yujian Z, Ling L, Yanying G, Kaiti X (2007). Waist-to-height ratio is an accurate and easier index for evaluating obesity in children and adolescents. Obesity.

[22] Ma GS, Ji CY, Ma J, Mi J, Yt Sung R, Xiong F, Yan WL, Hu XQ, Li YP, Du SM (2010). Waist circumference reference values for screening cardiovascular risk factors in Chinese children and adolescents. Biomed Environ Sci.

[23] Xu J, Long JS (2005). Confidence intervals for predicted outcomes in regression models for categorical outcomes. Stata J.

[24] Perneger TV (1998). What’s wrong with Bonferroni adjustments. Br Med J.

[25] StataCorp (2015). Stata Statistical Software: Release 14.1.

[26] Chagin AS, Savendahl L (2007). Estrogens and growth: review. Pediatr Endocrinol Rev.

[27] Lee JM, Wasserman R, Kaciroti N, Gebremariam A, Steffes J, Dowshen S, Harris D, Serwint J, Abney D, Smitherman L (2016). Timing of puberty in overweight versus obese boys. Pediatrics.

[28] Lee JM, Kaciroti N, Appugliese D, Corwyn RF, Bradley RH, Lumeng JC (2010). Body mass index and timing of pubertal initiation in boys. Arch Pediatr Adolesc Med.

[29] Crocker MK, Stern EA, Sedaka NM, Shomaker LB, Brady SM, Ali AH, Shawker TH, Hubbard VS, Yanovski JA (2014). Sexual dimorphisms in the associations of BMI and body fat with indices of pubertal development in girls and boys. J Clin Endocrinol Metab.

[30] Brambilla P, Manzoni P, Sironi S, Simone P, Del Maschio A, di Natale B, Chiumello G (1994). Peripheral and abdominal adiposity in childhood obesity. Int J Obes Relat Metab Disord.

[31] Caprio S, Hyman LD, McCarthy S, Lange R, Bronson M, Tamborlane WV (1996). Fat distribution and cardiovascular risk factors in obese adolescent girls: importance of the intraabdominal fat depot. Am J Clin Nutr.

[32] Bacopoulou F, Efthymiou V, Landis G, Rentoumis A, Chrousos GP (2015). Waist circumference, waist-to-hip ratio and waist-to-height ratio reference percentiles for abdominal obesity among Greek adolescents. BMC Pediatr.

[33] Kuba VM, Leone C, Damiani D (2013). Is waist-to-height ratio a useful indicator of cardio-metabolic risk in 6-10-year-old children?. BMC Pediatr.

[34] Freedman DS, Serdula MK, Srinivasan SR, Berenson GS (1999). Relation of circumferences and skinfold thicknesses to lipid and insulin concentrations in children and adolescents: the Bogalusa heart study. Am J Clin Nutr.

[35] Frisch RE, Revelle R (1970). Height and weight at menarche and a hypothesis of critical body weights and adolescent events. Science.

[36] Karapanou O, Papadimitriou A (2010). Determinants of menarche. Reprod Biol Endocrinol.

[37] Chen K, Corpus D, Zhong C, Rabii K, Klein K (2016). Ethnicity and excess body weight impact on pubertal onset in girls: a longitudinal study of hormonal and bone maturation changes. J Pediatr Endocrinol.

